# Data for Decision Makers: Finding Policy, Systems, and Environmental Solutions for Public Health Problems

**DOI:** 10.5888/pcd21.240165

**Published:** 2024-06-13

**Authors:** Deborah A. Galuska, Janet E. Fulton, LaToya J. O’Neal

**Affiliations:** 1 Division of Nutrition, Physical Activity, and Obesity, National Center for Chronic Disease Prevention and Health Promotion, Centers for Disease Control and Prevention, Atlanta, Georgia; 2 University of Florida, Institute of Food and Agricultural Sciences, Department of Family, Youth and Community Sciences, Gainesville, Florida

## Introduction

Public health decision makers are tasked with developing solutions to a variety of health problems. Policy, systems, and environmental (PSE) approaches are part of the portfolio of options they can use (1,2). PSE approaches work at a macro level and aim to improve health by changing factors such as rules or laws (policy), organizational procedures or protocols (systems), or physical, social, and economic environments (1).

PSE approaches are important for several reasons (1,2). First, because they operate at the macro level, PSE approaches can affect large numbers of people (1,2). For example, US Department of Agriculture (USDA) policies about what can be served in school lunches affect all children in schools who receive federal funding for their lunch programs. Second, they can limit people’s ability to engage in an unhealthy behavior or make it easier for people to choose healthy options; this influence is at a larger scale than individual behavior-change interventions (2). For example, workplace smoke-free policies that preclude workers from smoking for large parts of the day reduce tobacco use (3) and built environment interventions that improve routes such as sidewalks to everyday destinations make it easier for people to be physically active (4). Third, PSE approaches potentially have longer term sustainability (1), in part because they can become institutionalized or more permanent. For example, once sidewalks exist, they are harder to eliminate than a physical activity program in a local community center. Finally, PSE strategies have the potential to improve health equity by addressing root causes of health disparities (1). These root causes can include economic stability, educational access and quality, health care access and quality, neighborhood and built environments, and social and community contexts (5,6). Addressing these root causes has the potential to address multiple health outcomes (6).

Decision makers are influenced by many factors when deciding on a PSE strategy, including needed partnerships, political or leadership will, community acceptance, and feasibility (7). However, information is also important for decision making; evidence from various sources, including local data, also influences decision makers (7). Brownson and colleagues identified 3 potential points where information could have influence: policy process, policy content, and policy outcome (8). Health scientists, including data scientists, epidemiologists, researchers, and evaluators, have an important role in providing this information by answering questions such as:

- What health problems need PSE solutions?
- What PSE solutions should be considered?
- What is the uptake of PSE solutions?
- What is needed to successfully implement PSE solutions in the real world?

In this guest editorial, we explore how research, surveillance, and evaluation can be used to answer these key questions by using examples from this special *Preventing Chronic Disease* collection: Policy, Systems, and Environmental Approaches in Chronic Disease Research and Practice. We will also propose 4 additional opportunities for health scientists to advance implementing PSE solutions.

## What Public Health Problems Need PSE Solutions?

PSE solutions can be used to address most public health problems. However, because they can affect large numbers of people, they can be particularly considered for solving problems that also affect large numbers of people, for example, a health condition or risk factor with high population-wide prevalence. The article by Rippin et al illustrates how data can be used to identify such a problem (9). The authors used data collected from the standardized and nationally representative World Health Organization (WHO) Steps Survey to document the prevalence of fruit and vegetable consumption across 9 countries in the WHO European Region. The authors reported a high prevalence of inadequate fruit and vegetable consumption across all countries, ranging from 60% to 88%. Although consumption generally increased with education, it remained relatively low across most educational groups. The extensive and elevated prevalence of this risk factor for multiple chronic diseases suggests that countries and groups helping these countries should consider PSE solutions to address the problem. Examples could include interventions that improve the availability or affordability of fruits and vegetables (10).

In addition to identifying existing problems that might benefit from PSE solutions, research can also be used to identify new potential targets for PSE interventions. For example, Voyer et al used data from the 2020 Behavioral Risk Factor Surveillance System to characterize potential risk factors for subjective cognitive decline (SCD) (11). The authors highlighted a positive association between 2 or more adverse childhood experiences (ACES) and SCD, identifying ACES as a potentially modifiable risk factor for SCD. If a causal relationship is established, PSE interventions that address ACES, such as improving family economic security (12), are a potential tool for preventing cognitive decline.

## What PSE Solutions Should Be Considered?

Once decision makers identify areas for PSE interventions, they need to know what evidence-based PSE strategies are available. Groups such as the Community (13) or the US (14) Preventive Services Task Forces use rigorous methods to recommend evidence-based PSE solutions for communities or clinical settings that address public health problems. Often many PSE options can be considered for a single health problem. For example, Voyer et al (11), in their article on SCD, provide a table with examples of PSE interventions that could be used to address modifiable risk factors for SCD including built environment interventions for physical inactivity.

## What Is the Uptake of PSE Solutions?

Once decision makers identify and recommend a PSE strategy, they need to learn about its use. They need to know whether the recommended PSE strategy is adopted and, if so, by what groups, with what speed, and how well it is executed.

Tracking the implementation of PSE strategies can occur nationally to inform the decisions of multiple partners. Articles by Webber et al (15) and Onufrak et al (16) illustrate this by using data from the Community-Based Survey of Supports for Healthy Eating and Active Living (CBS-HEAL), a nationally representative survey of US municipalities. They documented the proportion of communities implementing recommended policies and practices that support physical activity, diet, and breastfeeding. They also documented changes in these policies between 2014 and 2021 and whether changes varied by community characteristics. For example, Webber et al found that the most common physical activity policy was maintenance for green spaces and equipment (86%) and that Complete Streets policies increased more between 2014 and 2021 for communities with larger versus smaller population sizes (15). Onufrak et al found that policies that support farmers markets were common (60%) and that the largest change in nutrition policies was for breastfeeding breaktime for government employees (an increase of 27 percentage points) (16). Data from surveillance such as CBS-HEAL can be used by funders of communities (eg, Centers for Disease Control and Prevention or US Department of Transportation) to determine whether the PSE strategies they promote are being implemented and identify where additional resources or technical assistance is needed to maximize impact.

Tracking can also occur at a program level to inform the decisions of the program funders. The article by Velarde et al demonstrates the uptake and impact of PSE interventions as part of the USDA-funded SNAP-Ed program (Supplemental Nutrition Assistance Program Education) (17). The authors used a standardized measurement tool to examine the impact over 4 years of an intervention in New Mexico to help schools implement at least 1 PSE strategy for nutrition and physical activity. They found significant improvements in school nutrition but not physical activity policies and environments. In this real-world setting, PSE interventions varied among the 11 elementary schools assessed, illustrating the evaluation challenge of balancing findings from community-driven interventions with obtaining generalizable findings.

## What Is Needed to Successfully Implement PSE Solutions in the Real World?

When PSE interventions are slow to be adopted or do not work as intended, they cannot have their intended impact. Decision makers need to understand why so they can provide supports for success.

The article by Wood et al illustrates how this information can be obtained (18). The authors conducted a comprehensive mixed-methods evaluation to identify facilitators and barriers to adopting a food service guidelines policy in Los Angeles County. They specifically examined how nutrition standards and practices were integrated into food service contracts in county government departments over a 10-year period, from 2011 through 2021. Facilitators identified included understanding the contracting process in the departments affected, building relationships with affected departments, designing guidelines and standards that could meet everyone’s needs, and providing tools and technical assistance to those implementing policies. Barriers included the complexity of the contracting environment and lack of resources and technical expertise on nutrition in the departments needed to effectively implement the policy. These lessons are likely not specific to implementing food service guidelines and underscore the need for strategic planning when starting PSE strategies.

## Potential Future Actions

The articles in this collection illustrate some ways health scientists can provide information that decision makers need to recommend PSE solutions for major public health problems. We propose 4 future actions health scientists could take to advance the implementation of PSE solutions.

**Recognize the value of community engagement and incorporate it into the work.** Community engagement is defined as the “the process of working collaboratively with and through groups of people affiliated by geographic proximity, special interest, or similar situations to address issues affecting the well-being of those people” (19). Community engagement gives voice to community members, particularly those not often heard, to actively create and influence solutions to problems affecting them (20). This engagement can help ensure that PSE strategies are responsive to the needs of the community, supported by the community, and culturally appropriate.

Many expert groups document and recognize the importance of community engagement in developing public health interventions and research (5,19–22). Proposed benefits of community engagement include improved trust, a better understanding of causes of a problem, improved community capacity to implement solutions, more practical and feasible solutions, and acceptance of the final solution (23). Community engagement can occur as part of planning, the interpretation of findings or data, or evaluation.

Health scientists, using their experience in data collection, can help decision makers obtain this input from community members. They can also incorporate community engagement in their own research and evaluation of PSE strategies using established tools and frameworks (20,21,24).

**Improve measurement of PSE indicators.** To assess the presence and use of PSE interventions, accurate information is needed. However, obtaining this information can be challenging, particularly when gathering this information across many groups, as is done in surveillance. For example, information on policies is often found in detailed government documents that might be time-consuming to find or hard to interpret. To improve timeliness, large surveys often use self-reported data. However, the validity may not be known. Studies that document the validity of different methods of capturing PSE interventions would ensure decisions are made from accurate assessments. The advance of artificial intelligence can potentially streamline finding policies and interpreting them to facilitate assessment.

Because implementation of PSE strategies often takes time, another avenue for research is identifying measures that align to different stages of the implementation process. Such information would allow funders to capture early measures of success and implementors to identify early stages for intervention. For example, Wood et al identified 4 phases of the food service guidelines contracting process where these types of measures could be incorporated (18).

**Provide additional information and tools that strengthen the real-world implementation of PSE strategies.** Decision makers need information on how to implement PSE strategies in real-world settings, and health scientists can do additional work to provide this information. The field of implementation science provides frameworks to guide this work (25). Tools and supports that effectively translate findings from this work could also help decision makers and practitioners apply it practically. For example, in addition to identifying barriers and facilitators to implementation, researchers can determine the necessary components of quality PSE interventions (8). By knowing what components of research-tested interventions must be kept versus what components can be adapted to community context, decision makers and implementors can design effective strategies from the beginning. Researchers can also develop tools that help others assess the quality of policies. Examples of these tools include those developed to measure the quality of policies for Complete Streets (26) and food service guidelines (27). Health scientists are also encouraged to share findings even when the PSE strategy was less effective than intended; lack of statistical significance does not mean findings are not useful.

**Determine whether PSE interventions have unintended outcomes and why.** PSE approaches are often chosen because they can benefit many people. However, unintentional consequences can occur in which not everyone benefits. For example, strategies to make communities more walkable can potentially increase property values, forcing lower-income community members to move and not benefit from the improvements (28).

Health scientists can design evaluations that assess the effect of policies in different types of communities or population groups, particularly those that are historically disadvantaged. In addition to documenting whether intended outcomes are equitably achieved, they can also consider whether unintended consequences occurred — for whom and why. Examples of unintended consequences that could be assessed include adverse economic effects, increased disparities in access to resources such as quality education or health care, or deleterious changes in other health outcomes.

## Conclusion

PSE strategies are an important tool with the potential to provide substantial and sustainable improvements to public health problems. However, decision makers need actionable information to select the most appropriate PSE interventions and to determine when and how to best implement them. Health scientists have an important role in providing that information.
